# Pharmacological Actions of 5-Hydroxyindolin-2 on Modulation of Platelet Functions and Thrombus Formation via Thromboxane A_2_ Inhibition and cAMP Production

**DOI:** 10.3390/ijms232314545

**Published:** 2022-11-22

**Authors:** Hyuk-Woo Kwon, Sung Dae Kim, Man Hee Rhee, Jung-Hae Shin

**Affiliations:** 1 Department of Biomedical Laboratory Science, Far East University, Eumseong 27601, Republic of Korea; 2 Department of Microbiological Resource Research Institute, Far East University, Eumseong 27601, Republic of Korea; 3 Department of Veterinary Medicine, College of Veterinary Medicine, Kyungpook National University, Daegu 41566, Republic of Korea; 4 Cardiovascular Research Institute, School of Medicine, Kyungpook National University, Daegu 41944, Republic of Korea

**Keywords:** 5-hydroxyindolin-2-one, *Protaetia brevitarsis* larvae, αIIbβ3 action, granule secretion, clot retraction

## Abstract

Platelets play a very significant role in hemostasis while simultaneously posing a risk for the development of various cardiovascular diseases. Platelet-mediated issues can occur in blood vessels and trigger various medical problems. Therefore, controlling platelet function is important in the prevention of thrombosis. In this regard, we need to find compounds that provide potent antiplatelet activity with minimum side effects. Therefore, we examined the effect of 5-hydroxyindolin-2-one isolated from *Protaetia brevitarsis* larvae having antiplatelet properties and investigated different pathways that mediate the antiplatelet activity. We examined the effect of 5-hydroxyindolin-2-one (5-HI) on the regulation of phosphoproteins, thromboxane A_2_ generation, and integrin αIIbβ3 action. Our data showed that human platelet aggregation was inhibited by 5-HI (75, 100, 150, 200 μM) without cytotoxicity, and it suppressed intracellular Ca^2+^ concentration through the regulation of inositol 1, 4, 5-triphosphate receptor I (Ser^1756^) and extracellular signal-regulated kinase (ERK). Moreover, collagen-elevated thromboxane A_2_ production and αIIbβ3 action were inhibited by 5-HI through the regulation of cytosolic phospholipase A_2_ (cPLA_2_), mitogen-activated protein kinase p38 (p38^MAPK^), vasodilator-stimulated phosphoprotein (VASP), phosphoinositide 3-kinase (PI3K), and Akt (protein kinase B). Therefore, we suggested that 5-HI could be a potential substance for the prevention of thrombosis-mediated thrombosis.

## 1. Introduction

Cardiovascular disease (CVD), which affects the heart and blood vessels, is the leading cause of death worldwide [1]. CVD includes various diseases such as atherosclerosis, coronary heart disease, heart attack, heart failure, and stroke. Among the 18.6 million CVD deaths worldwide in 2019, 58% occurred in Asia [2]. The Korean Society of Cardiology has recently published the Korea Heart Disease Fact Sheet 2020 and showed that CVD mortality has increased during the last decade in Korea [3]. In addition, many studies are being conducted in Korea to investigate the relationship between cardiovascular disease and other diseases and various lifestyles. Among several risk factors of CVD, platelet mediated thrombosis is one of the most important factors, and various antiplatelet drugs are used to improve cardiovascular disease [4]. Under normal circulatory conditions, platelets can play a vital role in maintaining hemostasis; however, aberrant platelet activation may cause thrombosis and atherosclerosis [5]. Platelet aggregation in the blood vessels is central to the development of thrombosis; thus, the regulation of the platelet is important in preventing cardiovascular issues. Therefore, current antithrombosis therapies target platelet inhibition pathways [6]. Collagen from damaged blood vessel activates platelets hydrolyze membrane phospholipids to inositol 1, 4, 5-trisphosphate (IP_3_), and IP_3_ is liberated into the cytoplasm. [Ca^2+^]_i_ mobilization from endoplasmic reticulum is controlled by IP_3_ receptor type I (IP_3_RI) on the surface of endoplasmic reticulum. At the same time, depletion of the [Ca^2+^]_i_ level causes Ca^2+^ influx, and extracellular signal-regulated kinase (ERK) regulates the influx [7,8]. Elevated [Ca^2+^]_i_ level facilitates granule release, and activated platelets produce thromboxane A_2_ (TXA_2_), leading to thrombus formation [9]. These actions finally activate glycoprotein IIb/IIIa (αIIb/β_3_) after signal transduction processes, and αIIb/β_3_ leads to the formation of platelet meshes at the site of damage to blood vessels [10,11].

Insects have been considered as drug resources, and many insects are being used in Oriental medicine. Among the various insect resources, *Protaetia brevitarsis* larvae is considered to be an important source of medicinal substance. Recently, the *Protaetia brevitarsis* larvae have also been approved by the Ministry of Food and Drug Safety as a food resource in Korea [12]. In order to verify the effect of *Protaetia brevitarsis* larvae, research on the component analysis has been conducted and alkaloid components were discovered [13]. Among the alkaloids were 5-hydroxyindolin-2-one (5-HI) and (1R,3S)-1-methyl-1,2,3,4-tetrahydro-b-carboline-3-carboxylic acid inhibited in vitro U46619-, and collagen-stimulated human platelet aggregation, coagulation activity, and bleeding time [14]. However, the detailed inhibitory mechanism has not been studied. Therefore, we used 5-HI to evaluate the effect on the three activation mechanisms of platelets and to determine what works by inhibiting them. In this study, we examined whether 5-HI inhibits [Ca^2+^]_i_ level, thromboxane A_2_ generation, and αIIb/β_3_-induced thrombus formation through the associated signaling molecules.

## 2. Results

### 2.1. 5-HI Blocks Platelet Activity, Cytotoxicity, and Half Maximal Inhibitory Concentration (IC_50_)

**Figure 1:**
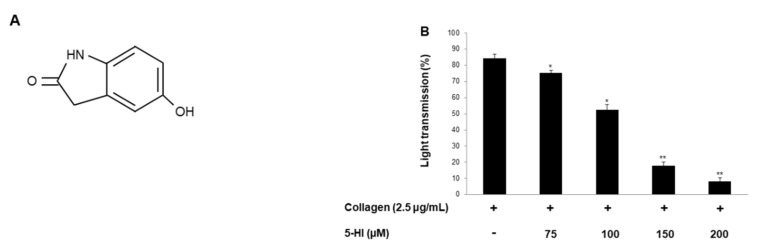

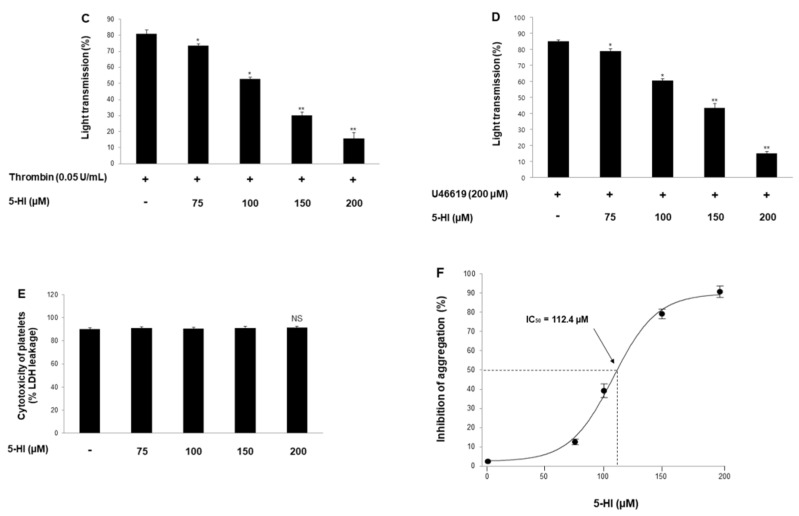
Effect of 5-HI on platelet aggregation. (**A**) Chemical structure of 5-HI (5-hydroxyindolin-2-one, MW. 149.15). (**B**) 5-HI’s effect on collagen-induced human platelet aggregation. (**C**) 5-HI’s effect on thrombin-induced human platelet aggregation. (**D**) 5-HI’s effect on U46619-induced human platelet aggregation. (**E**) 5-HI’s effect on cytotoxicity. (**F**) Half maximal inhibitory concentration (IC_50_) value of 5-HI in collagen-induced human platelet aggregation. Platelet aggregation and cytotoxicity were carried out as described in “Materials and Methods” section. The data are expressed as the mean ± standard deviation (n = 4). * *p* < 0.05, ** *p* < 0.01 versus each agonist-stimulated human platelets. NS—not significant.

To evaluate the antiplatelet activity of 5-HI (Figure 1A), various agonists were used for platelet aggregation. Collagen (2.5 μg/mL)-, thrombin (0.05 U/mL)-, and U46619 (200 nM)-activated platelets formed maximal aggregation. However, 5-HI inhibited all agonists-induced platelet aggregation (Figure 1B–D), and among them, collagen-induced aggregation was the most strongly inhibited. The 5-HI treated platelets showed no cytotoxicity (Figure 1E), and IC_50_ was 112.4 (Figure 1F).

### 2.2. 5-HI Blocks [Ca^2+^]_i_ Levels, IP_3_RI-, ERK-Phosphorylation, and Granule Release

**Figure 2:**
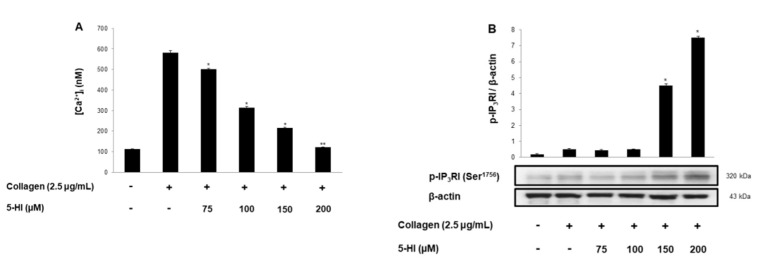

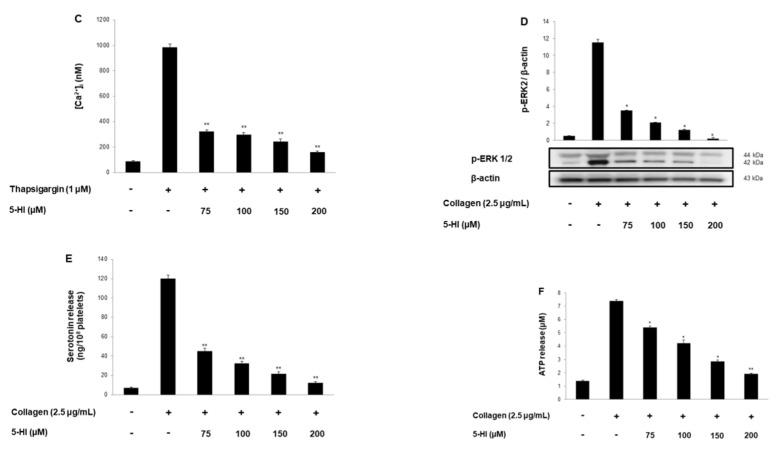
Effect of 5-HI on [Ca^2 +^ ]_i_ mobilization, Ca^2+^ influx, IP_3_RI and ERK phosphorylation, and serotonin and ATP release. (**A**) Effect of 5-HI’s effect on collagen-induced [Ca^2+^]_i_ mobilization. (**B**) 5-HI’s effect on thapsigargin-induced Ca^2+^ influx. (**C**) 5-HI’s effect on collagen-induced IP_3_RI phosphorylation. (**D**) 5-HI’s effect on collagen-induced ERK phosphorylation. (**E**) 5-HI’s effect on serotonin release. (**F**) 5-HI’s effect on ATP release. All experiments were performed as described in “Materials and Methods” section. The data are expressed as the mean ± standard deviation (n = 4). * *p* < 0.05, ** *p* < 0.01 versus the collagen-stimulated human platelets.

Next, intracellular calcium concentration and calcium-associated signaling molecules were evaluated. Collagen-induced intracellular calcium levels ([Ca^2+^]_i_) were elevated to 582.5 ± 8.2 nM, but 5-HI treated [Ca^2+^]_i_ mobilization was strongly inhibited (Figure 2A). It is well-known that cAMP/cGMP-dependent kinases phosphorylate IP_3_RI and its phosphorylation lead to the inhibition of [Ca^2+^]_i_ mobilization [15]. Therefore, we investigated whether 5-HI can influence IP_3_RI phosphorylation, and we confirmed that 5-HI showed strong IP_3_RI phosphorylation (Figure 2B). Another pathway is Ca^2+^ influx, which also increases the [Ca^2+^]_i_ level. Thus, we evaluated whether 5-HI can affect thapsigargin-induced Ca^2+^ influx via ERK phosphorylation. As shown in Figure 2C,D, thapsigargin-induced Ca^2+^ influx was suppressed by 5-HI through ERK phosphorylation [16,17].

Elevated [Ca^2+^]*_i_* concentration can facilitate the phosphorylation of myosin light chain and pleckstrin to trigger granule release. Therefore, we evaluated serotonin and ATP release from δ-granule. As shown in Figure 2E,F, 5-HI inhibited collagen-stimulated serotonin and ATP release dose-dependently.

### 2.3. 5-HI Blocks Thromboxane B_2_ Production and Dephosphorylation of cPLA_2_, p38^MAPK^

**Figure 3:**
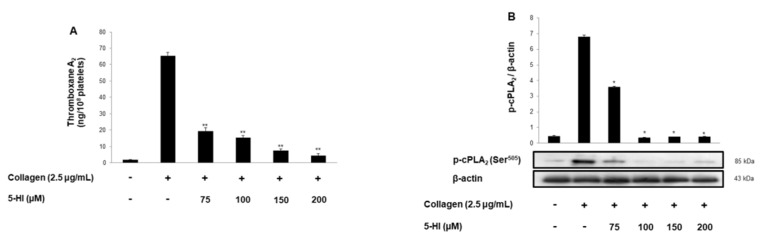

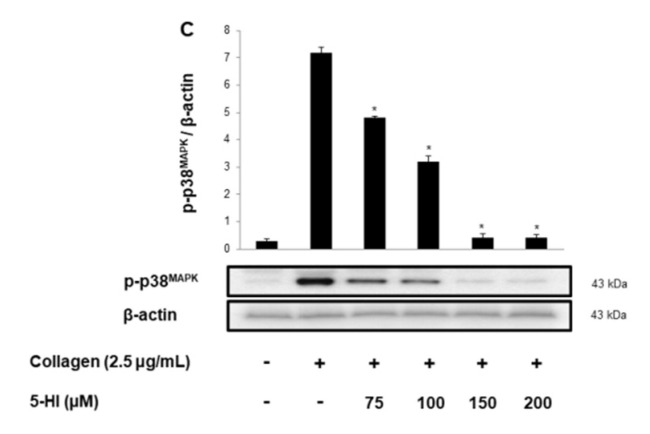
Effect of 5-HI on TXA_2_ production and cPLA_2_ and p38^MAPK^ phosphorylation. (**A**) 5-HI’s effect on collagen-induced TXA_2_ generation. (**B**) 5-HI’s effect on collagen-induced cPLA_2_ phosphorylation. (**C**) 5-HI’s effect on collagen-induced p38^MAPK^ phosphorylation. All experiments were performed as described in “Materials and Methods” section. The data are expressed as the mean ± standard deviation (n = 4). * *p* < 0.05, ** *p* < 0.01 versus the collagen-stimulated human platelets.

The synthesis of prostaglandin G_2_ and prostaglandin H_2_ from arachidonic acid is promoted through cyclooxygenase-1, and prostaglandin H_2_ is changed into TXA_2_. The TXA_2_ is a positive feedback mediator produced following platelet activation and acts as an agonist through Gq-coupled TXA_2_ receptor [18]. As shown in Figure 3A, collagen stimulation leads the TXA_2_ generation, but the production is inhibited by 5-HI. Next, we investigated TXA_2_-related signaling molecules such as cPLA_2_ and p38^MAPK^. As shown in Figure 3B,C, collagen-elevated cPLA_2_ and p38^MAPK^ phosphorylation were inhibited by 5-HI.

### 2.4. 5-HI Blocks Fibronectin Adhesion, Fibrinogen Binding, VASP, PI3K, and Akt Phosphoryation

**Figure 4:**
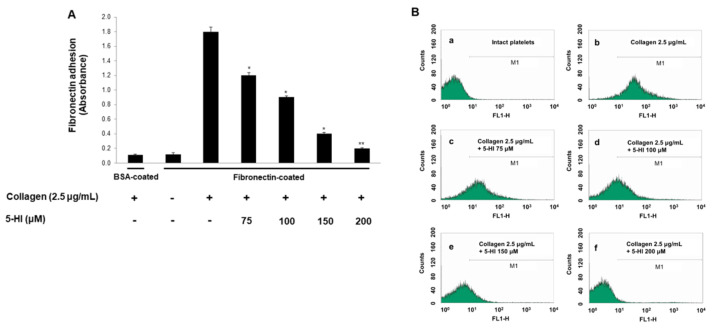

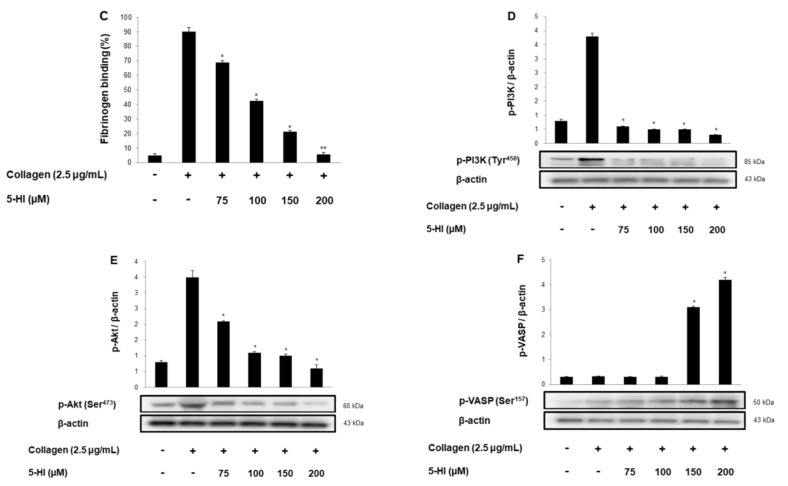
Effect of 5-HI on fibronectin adhesion, fibrinogen binding to αIIb/β3, and PI3K/Akt/VASP phosphorylation. (**A**) 5-HI’s effect on collagen-induced fibronectin adhesion. (**B**) The flow cytometry histograms on fibrinogen binding. (**a**), intact platelets (base); (**b**), collagen (2.5 μg/mL); (**c**), collagen (2.5 μg/mL) + 5-HI (75 μM); (**d**), collagen (2.5 μg/mL) + 5-HI (100 μM); (**e**), collagen (2.5 μg/mL) + 5-HI (150 μM); (**f**), collagen (2.5 μg/mL) + 5-HI (200 μM). (**C**) 5-HI’s effect on collagen-induced fibrinogen binding (%). (**D**) 5-HI’s effect on collagen-induced PI3K (Tyr^458^) phosphorylation. (**E**) 5-HI’s effect on collagen-induced Akt (Ser^473^) phosphorylation. (**F**) 5-HI’s effect on collagen-induced VASP (Ser^157^) phosphorylation. All experiments were performed as described in “Materials and Methods” section. The data are expressed as the mean ± standard deviation (n = 4). * *p* < 0.05, ** *p* < 0.01 versus the collagen-stimulated human platelets.

αIIb/β3 is the essential tool of platelet adhesion, binding, and spreading and causes platelet–platelet interaction and thrombus formation. The αIIb/β3 can bind to various adhesion proteins such as fibronectin and fibrinogen [10]; thus, we examined whether 5-HI-treated platelets can influence fibronectin adhesion. As shown in Figure 4A, the adhesion between platelets and fibronectin was inhibited by 5-HI. Next, we evaluated fibrinogen-binding action. Collagen induced the binding rate, which was 90.2 ± 2.5% (Figure 4Bb). However, 5-HI significantly decreased fibrinogen-binding action to αIIb/β3 (Figure 4Bc–f,C). Next, we investigated signaling molecules such as PI3K, Akt, and vasodilator-stimulated phosphoprotein (VASP) connected with αIIb/β3. PI3K and Akt are mediators in platelets, leading to platelet shape change and αIIb/β_3_ activation. The interaction between agonists and platelets facilitate class I PI3Ks, leading to the phosphatidylinositol 3,4,5 triphosphate accumulation. Then, Akt binds to phosphatidylinositol 3,4,5 triphosphate [19,20,21,22]. At the same time, VASP regulates actin for αIIb/β3 activation, but its phosphorylation inhibits actin elongation [23,24]. Our data showed that 5-HI significantly downregulated PI3K/Akt phosphorylation (Figure 4D,E) and upregulated VASP phosphorylation at Ser^157^ (Figure 4F).

### 2.5. 5-HI Elevated Cyclic Nucleotides and Suppressed Clot Retraction

**Figure 5:**
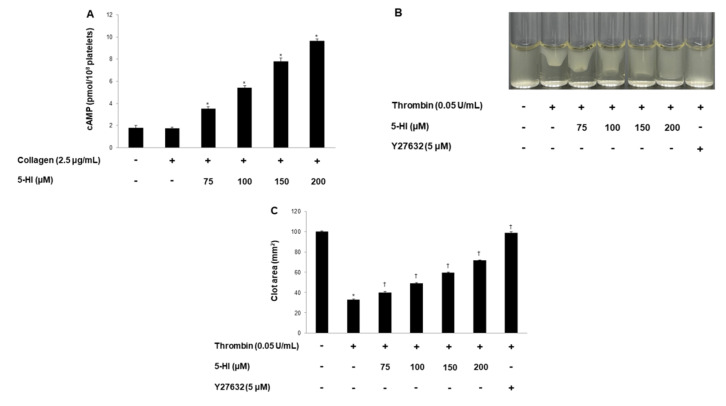
Effect of 5-HI on cAMP concentration and fibrin clot retraction. (**A**) 5-HI’s effect on collagen-induced cAMP production. (**B**) Photographs of fibrin clot. (**C**) 5-HI’s effect on thrombin-retracted fibrin clot (%). All experiments were performed as describe in “Materials and Methods” section. The data are expressed as the mean ± standard deviation (n = 4). * *p* < 0.05 versus the unstimulated human PRP, ^†^ *p* < 0.05 versus the thrombin-stimulated human PRP.

Next, we investigated cAMP and cGMP concentration in human platelets. Our result showed that 5-HI significantly increased cAMP levels (Figure 5A) but did not influence the cGMP level. Next, we investigated whether 5-HI affects fibrin clot retraction. As shown in Figure 5B, 5-HI effectively delayed clot formation, with inhibitory degrees of 60.6%, 50.8%, 40.5%, and 28.3%, respectively (Figure 5B,C). Y27632 was used as a positive control.

## 3. Discussion

Globally, cardiovascular diseases are considered among the leading causes of death. Thrombosis, a representative symptom of cardiovascular disease, is caused by an abnormal increase in thrombosis due to coagulation factors and platelet activation, which interferes with normal blood flow [25]. Antiplatelet agents, anticoagulants, and thrombolytic drugs are currently utilized for the prevention of thrombotic diseases. However, aspirin, an antiplatelet agent, has been reported to cause side effects such as gastrointestinal bleeding and ulceration [26]. Therefore, it is necessary to find new potential substances without side effects, and the candidate substances should be evaluated for their antiplatelet effect, anticoagulation effect, and thrombolytic activity. The current study of insects has been focused mainly on the value of edible insects as a new alternative resource for bioactive molecules. *Protaetia brevitarsis* larvae have traditionally been used in alternative medicine to cure toxic epilepsy, thrush, and tetanus [27]. Regarding the anticoagulant action, it has been reported that administration of *Protaetia brevitarsis* larvae ethanol extract showed antithrombotic efficacy in the rat [28], and insect-derived alkaloids prolonged activated partial thromboplastin time (APTT) and prothrombin time (PT) [14]. In addition, insect-derived alkaloids showed antiplatelet effects, and among them, we focused on 5-hydroxyindolin-2-one (5-HI) and conducted a study to identify the exact inhibitory mechanism.

The use of 5-HI suppressed various agonists-stimulated human platelet aggregation (Figure 1B–D) without cytotoxicity (Figure 2E). Next, we confirmed that 5-HI suppressed [Ca^2+^]_i_ levels. It is well-known that [Ca^2+^]_i_ levels are regulated by Ca^2+^ mobilization and Ca^2+^ influx, and the IP_3_RI and ERK phosphorylation is key for regulatory signaling molecules. Thus, we investigated whether 5-HI inhibited [Ca^2+^]_i_ levels through the phosphorylation of IP_3_RI and dephosphorylation of ERK. Our data showed that 5-HI strongly suppressed [Ca^2+^]_i_ levels through the phosphorylation of IP_3_RI and dephosphorylation of ERK (Figure 2A–D). Next, we examined whether 5-HI affects δ-granules release. Our data showed that collagen-stimulated serotonin and ATP secretion was inhibited by 5-HI (Figure 2E,F). Next, we determined that 5-HI inhibited TXA_2_ release (Figure 3A). Because TXA_2_ acts as a strong agonist, we focused on the TXA_2_ production and associated signaling molecules, such as cPLA_2_ and p38^MAPK^ [29]. We confirmed that 5-HI suppressed TXA_2_ production through the dephosphorylation of p38^MAPK^ and cPLA_2_ (Figure 3B,C).

Next, we investigated αIIb/β_3_ activation, leading platelet–platelet interaction. Various signaling events facilitate integrin activation, leading to the structural change of αIIb/β3. Activated platelets interact with other platelets via αIIb/β3, and as a result, another signaling mechanism begins inside the platelets. This signaling action is called the outside-in signaling pathway [30]. Regarding the activation of αIIb/β_3_, PI3K/Akt and VASP are the crucial mediators. Therefore, we investigated whether 5-HI suppresses αIIb/β3 action by the dephosphorylation of PI3K/Akt and phosphorylation of VASP (Ser^157^). The use of 5-HI suppressed αIIb/β3 affinity (Figure 4A–C), decreased PI3K/Akt phosphorylation (Figure 4D,E), and increased VASP phosphorylation (Ser^157^) (Figure 4F). The cAMP and cGMP act as negative feedback molecules within the platelet. The cAMP and cGMP are generated by adenylate and guanylate cyclase for antiplatelet function and are decomposed by phosphodiesterases [31,32]. These molecules can elevate the phosphorylation of VASP (Ser^157^, Ser^239^) and IP_3_RI (Ser^1756^). In our study, 5-HI increased only the cAMP level (Figure 5A), and these changes can block the αIIb/β3 action. Next, we investigated whether 5-HI can affect αIIb/β3-mediated fibrin clot retraction. As shown in Figure 5B,C, 5-HI strongly inhibited the clot retraction. Our study had some limitations: 5-HI increased the cAMP level in human platelets, and the antiplatelet effect of 5-HI is thought to be due to increased cAMP. However, 5-HI also affected the phosphorylation of p38^MAPK^ and cPLA_2_; this phosphorylation is not related to the increase in cAMP. Therefore, we could not clearly determine whether the antiplatelet effect of 5-HI is due to the increase in cAMP or if it can regulate all proteins in turn. Additionally, all of our studies were conducted in vitro. Although the antiplatelet effect of 5-HI is strong, it is difficult to predict the effect in the human body. The in vivo antiplatelet effect of *Protaetia brevitarsis* larvae ethanol extract has not been studied, and the in vivo bioactivity effect of 5-HI should also be evaluated in the future. However, 5-HI showed an effect in an in vivo bleeding test and delayed thrombus in an ex vivo coagulation test [14]. Therefore, based on the results, we suggest that 5-HI has the potential to inhibit thrombosis-mediated cardiovascular disease. Various laboratory data have shown that medicinal plants may have therapeutic potential in cardiovascular disease, and it has been identified that Ginseng, *Ginkgo biloba*, and *Ganoderma lucidum* have potential effects on cardiovascular disease in vitro and in vivo [33]. A randomized clinical trial was conducted on the use of Ginseng, *Ginkgo biloba*, and *Ganoderma lucidum* for the treatment of cardiovascular disease, but the therapeutic potential of medicinal plants in cardiovascular disease have not been clinically observed. However, coadministration of traditional cardiovascular disease drugs and natural products has shown potential for the inhibition of cardiovascular disease, and several clinical trials in cardiovascular disease are still ongoing [33]. In our previous study, ginsenosides showed strong antiplatelet effects, and their inhibitory concentrations were similar to those of 5-HI [34]. Therefore, 5-HI also has the potential for clinical application in patients with cardiovascular disease.

Beyond a crucial task in hemostasis and thrombosis, platelets are an important regulator of inflammatory reaction, immune response, atherosclerosis, and cancer metastasis. This is achieved by the expression of adhesive molecules and receptors on the platelet surface and by the release of secretory products including inflammatory mediators and cytokines [35]. Interactions between platelets and endothelial cells at atherosclerotic-prone sites can enhance the recruitment of leukocyte through the release of cytokines, chemokines, and proinflammatory molecules, and the interaction between platelets, endothelial cells, and leukocytes can promote a localized inflammatory response that can accelerate the early formation of atherosclerotic lesions [36,37]. In the pathogenesis of atherosclerosis, endothelial damage allows precipitation of low-density lipoprotein (LDL) to the subendothelial layer, and LDL are modified into oxidized LDL (ox-LDL). Platelets have receptors for ox-LDL, which can cause aggregation and form clots [38].

Looking at various studies related to the action of platelets, it has been reported that platelet mitochondrial reactive oxygen species contributes to age-related thrombosis, and endogenous superoxide dismutase 2 protects from platelet-dependent thrombin generation and thrombosis during aging [39]. Another report showed that platelets express pathogen-recognition molecules such as toll-like receptors and FcgRIIa receptor [40,41]. Therefore, platelets can become activated by an interaction with the pathogen, and it has also been reported that cancer cells can interact with platelets to cause platelet aggregation and tumor metastasis [42,43]. These various results show that platelets participate in various mechanisms and diseases. Therefore, the platelet aggregation reaction inhibited by 5-HI has a prospective potential in various clinical actions and diseases. We hope that 5-HI will be used in various research fields and be developed as a new natural drug.

**Figure 6:**
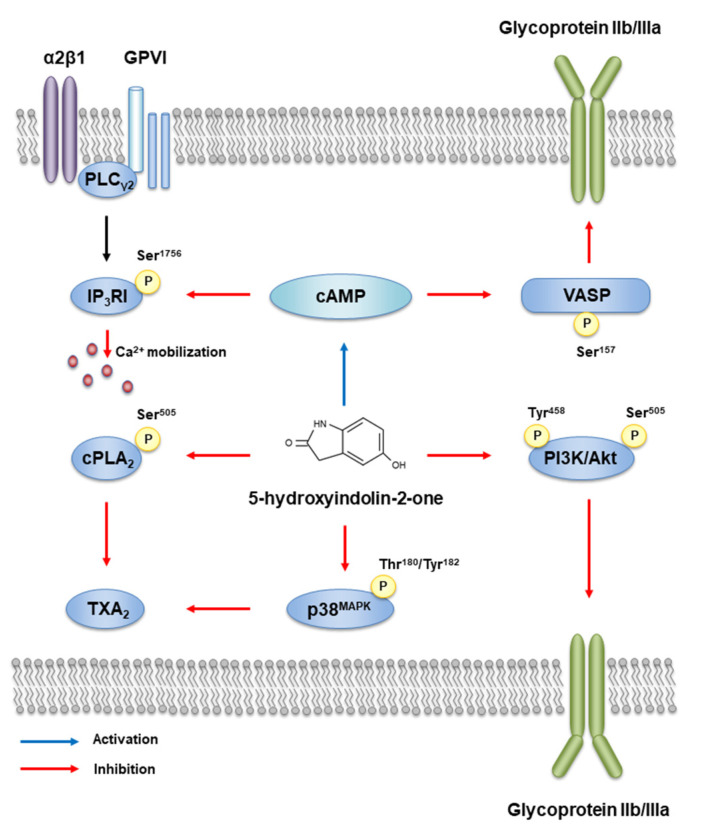
A schematic summary of inhibitory effects of 5-HI on platelet intracellular signaling pathway.

This study found that 5-HI decreased human platelet aggregation, calcium mobilization, fibronectin adhesion, fibrinogen binding, and clot retraction through the regulation of various phosphoproteins and cAMP. Therefore, 5-HI from *Protaetia brevitarsis* larvae would be a useful substance for the prevention of thrombosis. A summary of the inhibitory pathway and of the common effects of 5-HI on intracellular signaling by collagen-stimulated platelets is provided in Figure 6.

## 4. Materials and Methods

### 4.1. Materials

The supply of 5-hydroxyindolin-2-one (5-HI) was purchased from ChemFaces (Wuhan, China). Fura 2-AM (2-acetoxymethyl) and fibrinogen (Alexa Fluor 488 conjugated) were purchased from Invitrogen (Eugene, OR, USA). A serotonin detection kit was purchased from Labor Diagnostika Nord GmbH and Co. (Nordhorn, Germany). Physiological agonists (collagen, U46619, and thrombin) were obtained from Chrono-Log Co. (Havertown, PA, USA). Cell Signaling (Beverly, MA, USA) supplied anti-phospho-p38^MAPK^, anti-phospho-inositol-3-phosphate receptor type I (Ser^1756^), anti-phospho-ERK (1/2), anti-phospho-VASP (Ser^157^), anti-phospho-cPLA_2_ (Ser^505^), anti-phosphor-PI3K (Tyr^458^), anti-phospho-Akt (Ser^473^), anti-β-actin, and anti-rabbit secondary antibodies. The thromboxane B_2_, cAMP, and ATP detection kit were purchased from Cayman Chemical (Ann Arbor, MI, USA).

### 4.2. Human Platelet Aggregation

The human platelet-rich plasma (PRP) was obtained from the Korean Red Cross Blood Center (Suwon, Korea). The platelets were then washed twice with washing buffer and resuspended in suspension buffer. The platelet suspension was adjusted to a concentration of 10^8^/mL. Platelet suspensions (10^8^/mL) were preincubated with 5-HI at 37 °C for 5 min, and then collagen, thrombin, and U46619 were added for platelet activation. Platelet aggregation was measured for five minutes. The change in the light transmission was calculated as the aggregation rate (%), 5-HI was dissolved in 0.1% dimethyl sulfoxide (DMSO), and platelet aggregation was conducted using an aggregometer (Chrono-Log, Havertown, PA, USA).

### 4.3. Cytotoxicity Analysis

Cytotoxicity was assessed using lactate dehydrogenase (LDH) leakage assay. Platelets (10^8^/mL) were incubated with 5-HI for 20 min and incubated tubes were centrifuged at 12,000× *g* for supernatant. Lactate dehydrogenase was estimated using an ELISA reader (TECAN, Salzburg, Austria).

### 4.4. Ca^2+^ Analysis

The Fura 2-AM (5 μM) and human PRP were preincubated for 60 min at 37 °C, and platelet suspension was prepared. Platelets (10^8^/mL) were preincubated with 5-HI for 5 min at 37 °C and stimulated with collagen (2.5 μg/mL) for Ca^2+^ mobilization. For Ca^2+^ influx detection, platelets (10^8^/mL) were stimulated with thapsigargin (1 μM) in the presence of 100 μM of EGTA, and for thapsigargin stimulation, 2 mM of calcium was added at 3 min. The fluorescence was measured using a spectrofluorometer (Hitachi F-2700, Tokyo, Japan) (Hitachi F-2700, Tokyo, Japan), and the [Ca^2+^]_i_ values were calculated using the Grynkiewicz method [44].

### 4.5. Serotonin and ATP Analysis

Platelets (10^8^/mL) were preincubated for 5 min at 37 °C with 5-HI, and then stimulated with collagen (2.5 μg/mL) in the presence of 2 mM CaCl_2_ to terminate ATP release, followed by centrifugation. The supernatant was used for detection of ATP release. The ATP luminescent assay kit (Cayman Chemical, Ann Arbor, MI, USA) was detected using an ELISA reader (Tecan, Salzburg, Austria).

### 4.6. Thromboxane B_2_ Analysis

Because thromboxane A_2_ (TXA_2_) is quickly converted to thromboxane B_2_ (TXB_2_), TXA_2_ generation was measured by TXB_2_. After collagen-induced platelet aggregation with 5-HI, the reaction was terminated by indomethacin (0.2 mM), and the reaction tubes were centrifuged to obtain supernatant separation. TXB_2_ in the supernatant was detected using an ELISA reader (Tecan, Salzburg, Austria).

### 4.7. Immunoblotting Analysis

Collagen-induced platelet aggregation was terminated by adding lysis buffer, and proteins in the lysates were measured using a BCA protein assay kit (Pierce Biotechnology, Rockford, IL, USA). After sodium dodecyl sulfate polyacrylamide gel electrophoresis, proteins were transferred onto membranes and treated with primary and secondary antibodies. Detection was carried out in a dark room, and Western blotting bands were converted into a graph using the Quantity One program (Bio-Rad, Hercules, CA, USA).

### 4.8. Fibronectin Adhesion Analysis

Platelets (10^8^/mL) were preincubated with 5-HI and CaCl_2_ (2 mM) for 1 h at 37 °C in the presence of collagen (2.5 μg/mL) and washed twice with PBS buffer, followed by the addition of cell stain solution. Extraction solution was added after a washing step, and supernatant was placed onto a 96-well plate. The plate was read at a wavelength of 560 nm using an ELISA reader (Tecan, Salzburg, Austria).

### 4.9. αIIb/β3 Activity Analysis

Platelets (10^8^/mL) were preincubated with 5-HI, and collagen-induced platelet aggregation was conducted with Alexa Fluor 488-conjugated fibrinogen for 20 min. After aggregation, the reaction mixture was fixed with paraformaldehyde (0.5%). For the detection of αIIb/β3 activity, fixed platelet–fibrinogen was tested using flow cytometer (BD Biosciences, San Jose, CA, USA).

### 4.10. Fibrin Clot Retraction

For the fibrin clot retraction test, a human platelet-rich plasma (300 μL) was incubated with 5-HI for 30 min at 37 °C, and the clot reaction was triggered by thrombin (0.05 U/mL). After reacting for 15 min, pictures of fibrin clots were taken using a digital camera, and Image J (v1.46) was used for conversion to the clot area (National Institutes of Health, Bethesda, MD, USA).

### 4.11. Data Analyses

All data are presented as the mean ± standard deviation with various numbers of observations. To determine major differences among groups, analysis of variance was performed, followed by the Tukey–Kramer method. SPSS 21.0.0.0 software (SPSS, Chicago, IL, USA) was used for statistical analysis, and *p* < 0.05 was considered statistically significant.

## Data Availability

Not applicable.
